# Clinical applications of Impella 5.5 for acute severe bioprosthetic aortic regurgitation: a bridge to destination therapy report—case series and review

**DOI:** 10.1093/ehjcr/ytag573

**Published:** 2026-08-03

**Authors:** Ahmed Elshafie, Hussam Al Hennawi, Ahmad Abulshamat, Shaurya Srivastava, Rajit Pahwa, Appa Bandi, Hisham Qandeel, Mohammed Qintar

**Affiliations:** Department of Cardiology, University of Michigan Health—Sparrow Heart and Vascular, and Michigan State University, Lansing, MI 48912, USA; Geisinger College of Health Sciences, Department of Cardiology, Geisinger Wyoming Valley Medical Center, Wilkes-Barre, PA 18711, USA; Department of Cardiology, Jordan University Hospital, Amman 11942, Jordan; Department of Cardiology, University of Michigan Health—Sparrow Heart and Vascular, and Michigan State University, Lansing, MI 48912, USA; Department of Critical Care, University of Michigan Health, Lansing, MI 48912, USA; Department of Cardiology, University of Michigan Health—Sparrow Heart and Vascular, and Michigan State University, Lansing, MI 48912, USA; Department of Cardiology, University of Michigan Health—Sparrow Heart and Vascular, and Michigan State University, Lansing, MI 48912, USA; Department of Cardiology, University of Michigan Health—Sparrow Heart and Vascular, and Michigan State University, Lansing, MI 48912, USA; Department of Cardiology, Hamad Heart Hospital, Doha, Qatar

**Keywords:** Case series, Cardiogenic shock, Aortic insufficiency, Mechanical circulatory support, Transcatheter aortic valve replacement

## Abstract

**Background:**

Acute severe bioprosthetic aortic regurgitation complicated by cardiogenic shock represents a high-risk clinical scenario requiring urgent intervention. Conventional medical therapy is often insufficient, and options for mechanical circulatory support remain limited because severe aortic regurgitation is traditionally considered a relative contraindication to transvalvular support devices.

**Case summary:**

We describe two cases of acute severe bioprosthetic aortic regurgitation presenting with cardiogenic shock in which Impella 5.5 was utilized as temporary mechanical circulatory support to stabilize patients and facilitate definitive treatment. In the first case, Impella 5.5 support enabled successful bridge to valve-in-valve transcatheter aortic valve replacement. In the second case, Impella 5.5 was used as a bridge to redo surgical aortic valve replacement. In both cases, haemodynamic stabilization and end-organ recovery allowed progression to destination therapy.

**Discussion:**

These cases demonstrate that, in carefully selected patients with acute severe bioprosthetic aortic regurgitation and cardiogenic shock, Impella 5.5 may serve as a feasible bridge to definitive valve intervention. Although severe aortic regurgitation has traditionally limited the use of transvalvular mechanical support, these experiences suggest a potential role for Impella 5.5 in individualized management strategies. Further studies are needed to better define patient selection, safety, and outcomes.

Learning points

- Recognize the haemodynamic challenges and limited mechanical circulatory support options in patients with acute severe bioprosthetic aortic regurgitation complicated by cardiogenic shock.
- Understand the potential role of Impella 5.5 for left ventricular unloading and stabilization as a bridge to definitive valve intervention.
- Review multidisciplinary management strategies and procedural considerations for urgent valve-in-valve transcatheter aortic valve replacement or redo surgical valve replacement in high-risk patients.

## Introduction

Bioprosthetic aortic valves, whether implanted surgically or through transcatheter approaches, provide durable treatment for valvular heart disease but remain susceptible to structural valve deterioration over time. In some patients, degeneration may present acutely as severe bioprosthetic aortic regurgitation (AR), resulting in rapid haemodynamic deterioration and cardiogenic shock requiring urgent intervention.

Definitive treatment options include valve-in-valve (ViV) transcatheter aortic valve replacement (TAVR) and redo surgical aortic valve replacement (SAVR); however, patients frequently require temporary stabilization before these interventions can be safely performed. Selection of mechanical circulatory support (MCS) in this setting is challenging. Intra-aortic balloon pump (IABP) support is generally avoided because of the potential to increase regurgitant volume, while venoarterial extracorporeal membrane oxygenation (VA-ECMO) may increase afterload and worsen left ventricular distension if unloading is inadequate.

Although Impella support has traditionally been considered contraindicated or relatively contraindicated in severe AR because of concern for recirculation of forward flow across the incompetent aortic valve and ineffective unloading, emerging reports suggest that short-term Impella 5.5 support may be feasible in carefully selected patients when used as a bridge to definitive intervention and accompanied by close haemodynamic and imaging surveillance.

Here, we present two cases of acute bioprosthetic valve failure with severe AR complicated by cardiogenic shock in which Impella 5.5 was used as temporary support prior to definitive intervention. We describe the clinical rationale, haemodynamic response, and procedural considerations associated with this approach.

## Summary figure

**Figure:**
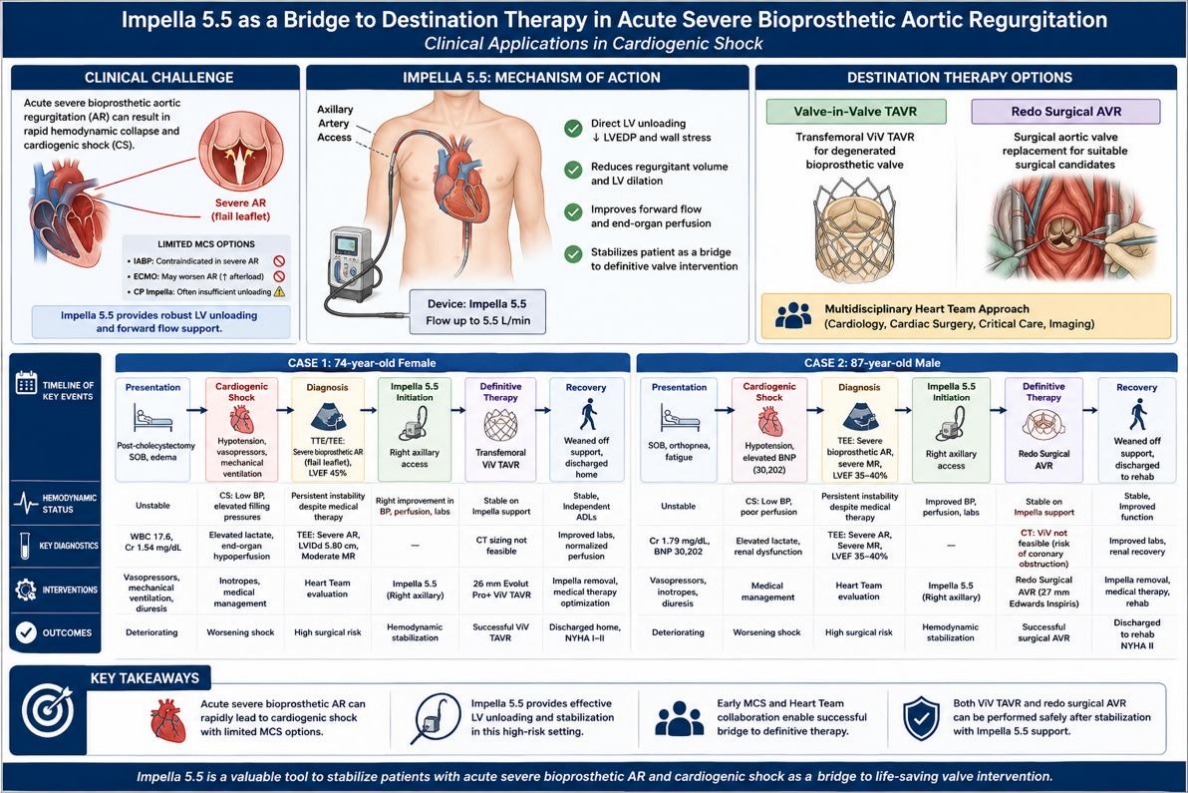


### Case 1

A 74-year-old woman with a history of paroxysmal atrial fibrillation on anticoagulation, moderate mitral regurgitation (MR), and prior bioprosthetic aortic valve replacement (27 mm Freestyle valve) with ascending aortic repair in 2010 presented with acute decompensated heart failure. She reported progressive shortness of breath and lower extremity swelling that developed shortly after discharge from a laparoscopic cholecystectomy performed 1 week prior. Although initially suspected to have septic shock, further evaluation revealed cardiogenic shock secondary to severe bioprosthetic AR, and sepsis was ruled out.

On examination, there were no signs of infection at her abdominal surgical site, and imaging ruled out intra-abdominal pathology. Laboratory tests showed an elevated creatinine level of 1.54 mg/dL (reference 0.6–1.2 mg/dL) and leucocytosis with white blood cell count 17.6 × 10^3^/µL (reference 4.0–11.0 × 10^3^/µL). She was initially started on vasopressor support and mechanically ventilated.

Transthoracic echocardiography (TTE) revealed a left ventricular ejection fraction (LVEF) of 45% with severe bioprosthetic regurgitation due to structural valve deterioration (flail leaflet) and moderate MR. Follow-up transoesophageal echocardiography (TEE) confirmed these findings and showed an MCS of 5.80 cm. The aortic valve demonstrated severe regurgitation with a mild paravalvular leak (peak velocity 1.36 m/s, peak gradient 7.4 mmHg, mean gradient 3.0 mmHg, and an aortic valve area of 1.69 cm^2^) (see Supplementary material online, *Videos S1 and S2*).

Despite medical management with inotropic support and diuresis, her haemodynamic status continued to decline. As a result, MCS with an Impella 5.5 device was initiated. Following right axillary Impella 5.5 implantation, objective haemodynamic and end-organ recovery was observed. On post-Impella day 1, Fick cardiac output and cardiac index improved to 6.6 and 3.4 L/min/m^2^ with cardiac power output of 1.05 W despite ongoing vasoactive support (milrinone 0.375 mcg/kg/min, noradrenaline 0.2 mcg/kg/min, and vasopressin 0.03 units/min). Pulmonary artery pressure (PAP) measured 57/25 mmHg (mean 36) with pulmonary artery pulsatility index (PAPI) of 4. By post-Impella day 2, cardiac index remained preserved at 3.3 L/min/m^2^ with reduction in PAP to 42/16 mmHg (mean 25), improvement in PAPI to 9, and pulmonary capillary wedge pressure (PCWP) of 12 mmHg. End-organ perfusion improved with lactate improved from 7.3 to 1.6 mmol/L (reference 0.5–2.0 mmol/L), urine output increasing from 185 mL pre-support to 780 and 910 mL over the subsequent 24-h periods, and creatinine improving from a peak of 2.2 mg/dL to 1.4 mg/dL prior to definitive valve intervention (*Table 1*).

**Table 1:** Objective haemodynamic, end-organ, and device parameters before and after Impella 5.5 Support

Parameter	Pre-Impella (cardiogenic shock)	Post-Impella Day 1	Post-Impella Day 2	Post-ViV TAVR
Mechanical support	None	Impella 5.5	Impella 5.5	Impella removed
Impella setting	N/A	P8	Continued support	Removed during procedure
Cardiac output (Fick)	Not available	6.6 L/min	6.6 L/min	Not available
Cardiac index (Fick)	Not available	3.4 L/min/m^2^	3.3 L/min/m^2^	Not available
Stroke volume	Not available	60 mL/beat	Not available	Not available
Cardiac power output	Not available	1.05 W	0.94 W	Not available
Pulmonary artery pressure	Not available	57/25 mmHg (mean 36)	42/16 mmHg (mean 25)	Not available
Pulmonary artery pulsatility index (PAPI)	Not available	4	9	Not available
Pulmonary capillary wedge pressure (PCWP)	Not available	Not available	12 mmHg	Not available
Systemic vascular resistance	Not available	776 dynes·sec/cm^5^	Not available	Not available
Milrinone	Required	0.375 mcg/kg/min	Not specified	Weaned off
Noradrenaline	Required	0.2 mcg/kg/min	Not specified	Weaned off
Vasopressin	Required	0.03 units/min	Not specified	Weaned off
Lactate	Peak 7.3 mmol/L	Improved to 1.8–1.2 mmol/L	2.1 → 1.6 mmol/L	Normalized
Urine output (24 h)	185 mL	780 mL	910 mL	Sustained improvement
Creatinine	Increased to peak 2.2 mg/dL (baseline 1.0)	Downtrending after support initiation	Improved to 1.4 mg/dL	Further improved to 1.31 mg/dL
BUN	Peak 72 mg/dL	Improving	Continued improvement	51 mg/dL
Total bilirubin	Peak 4.8 mg/dL	Improving	Improved to ∼2.0–2.3 mg/dL	Stable
Liver function	Mild transaminase elevation without progressive injury	Stable	Stable	Stable
Duration of Impella support	N/A	Ongoing	Ongoing	∼2 days total (bridge to emergent ViV TAVR)
Device-related complications	None	None	None	Complete heart block requiring permanent pacemaker

**Figure 1:**
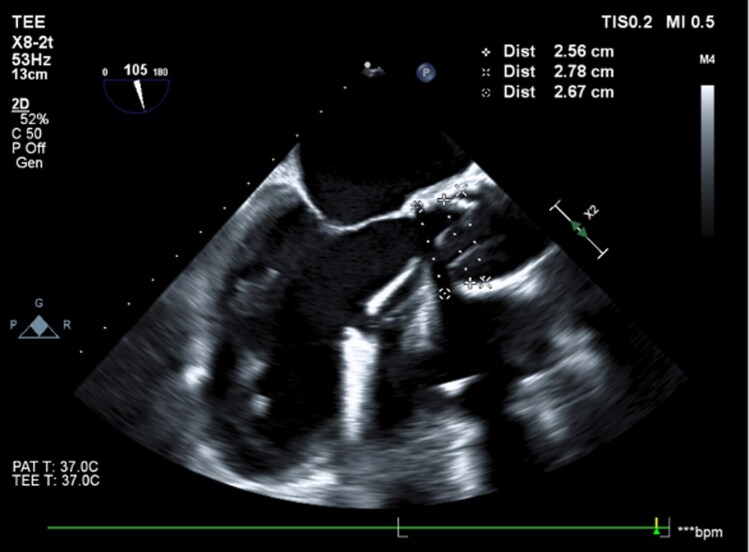
A transoesophageal echocardiogram image showing valve sizing on preprocedural transoesophageal echocardiogram.

Due to her critical state, pre-procedural imaging and CT-based valve sizing were not feasible. Valve selection was instead based on TEE imaging, which showed an aortic root measurement of ∼2.67 cm (*Figure 1*). Consequently, a 26 (+2) mm Edwards Sapien S3 Ultra valve was selected for the transcatheter ViV TAVR procedure. The risk of coronary obstruction, valve sizing, ViV feasibility, and surgical bailout was assessed through angiogram.

The TAVR was performed under angiographic and TEE guidance. During deployment, the Impella device was temporarily withdrawn into the ascending aorta and later removed. The valve was successfully deployed under rapid pacing at 200 beats per minute, with no valvular or paravalvular regurgitation observed on post-deployment TEE (see Supplementary material online, *Videos S3 and S4*).

Following the procedure, the patient developed a complete heart block with minimal escape rhythm, requiring permanent pacemaker implantation. Her haemodynamics quickly improved, allowing for weaning from vasopressor support and continued intravenous diuresis. She tolerated goal-directed medical therapy and was ultimately discharged to inpatient cardiac rehabilitation. At discharge, she was ambulatory and actively participating in her recovery. She was seen 1 month in the structural clinic, and she had returned entirely to being asymptomatic and in NYHA Class I without any symptoms.

### Case 2

A 63-year-old man with a complex cardiac history, including severe bicuspid aortic stenosis status post-SAVR with a 25 mm Medtronic Mosaic bioprosthetic valve 10 years prior, coronary artery disease status post mid-LAD stenting in 2014 following a myocardial infarction, hyperlipidaemia, obesity, obstructive sleep apnoea, and chronic obstructive pulmonary disease not requiring home oxygen, presented with acute chest pain and worsening dyspnoea following heavy lifting. He was also known to have an ascending aortic aneurysm.

On presentation, his vital signs revealed hypertension (blood pressure in the 160s mmHg), tachycardia (heart rate in the 100s bpm), and oxygen saturations of 90% on room air. Initial laboratory evaluation showed lactate 2.2 mmol/L (reference 0.5–2.0 mmol/L), high-sensitivity troponin peaking at 18 609 ng/L (reference <14 ng/L; specify assay if available), B-type natriuretic peptide 621 pg/mL (reference <100 pg/mL), and creatinine 1.32 mg/dL (reference 0.7–1.3 mg/dL). His workup was concerning for a non-ST-elevation myocardial infarction and pulmonary oedema, although a coronary angiogram demonstrated minimal coronary disease with a patent LAD stent, and the evaluation was negative for pulmonary embolism.

TTE revealed moderate-to-severe bioprosthetic AR with an LVEF of 40%–45%. Subsequent TEE confirmed a severely degenerated, calcified bioprosthetic AVR with severe AR and anteriorly directed severe MR secondary to a flail posterior leaflet (see Supplementary material online, *Videos S5 and S6*). Additionally, an ascending aortic aneurysm measuring 4.5 cm was identified.

The patient’s respiratory status deteriorated, requiring endotracheal intubation and mechanical ventilation. Inotropic support with milrinone was initiated. Given his acute decompensated state and complex valvular pathology, a multidisciplinary heart team decision was made to proceed with emergent redo surgical intervention.

To optimize preoperative haemodynamics and improve end-organ perfusion, an Impella 5.5 was implanted via right axillary artery cutdown on hospital day 6. Before support, invasive haemodynamics demonstrated severe cardiogenic shock with cardiac output of 3.1 L/min, cardiac index of 1.7 L/min/m^2^, PCWP of 40 mmHg, PAP of 67/39 mmHg (mean 49), RA pressure of 15 mmHg, stroke volume of 40 mL, and PAPI of 1.86 while receiving noradrenaline 0.05 mcg/kg/min. During 3 days of Impella support, cardiac output improved to 6.3–6.8 L/min with cardiac index approaching 2.9 L/min/m^2^, pulmonary pressures decreased, urine output increased from ∼400–1250 mL/day pre-support to a peak of 3.45 L/day, lactate normalized to 1.5–1.6 mmol/L, and renal function improved (creatinine 2.22 to 1.11 mg/dL), permitting bridge to definitive redo valve surgery. Despite successful surgical correction, severe postcardiotomy ventricular dysfunction necessitated escalation to VA-ECMO and subsequent Impella CP support (*Table 2*).

**Table 2:** Objective haemodynamic, end-organ, and device parameters before and after Impella 5.5 Support (Case 2)

Parameter	Pre-Impella (cardiogenic shock)	Post-Impella Day 1	Post-Impella Day 2	Post-Impella Day 3/pre-operative	Post-operative course
Mechanical support	None	Impella 5.5	Impella 5.5	Impella 5.5	Impella removed intraoperatively → VA-ECMO → Impella CP
Duration of Impella support	N/A	Ongoing	Ongoing	Total support: 3 days	Escalation due to postcardiotomy shock
Vasopressor/inotrope support	Noradrenaline 0.05 mcg/kg/min	Continued support	Continued support	Continued support	High-dose inotropes + ECMO
Cardiac output	3.1 L/min	4.9–5.8 L/min	6.3–6.8 L/min	Maintained >6 L/min	Low output postcardiotomy state
Cardiac index	1.7 L/min/m^2^	2.0–2.5 L/min/m^2^	2.7–2.9 L/min/m^2^	Peak ∼2.9 L/min/m^2^	Post-CPB severe LV dysfunction
Stroke volume	40 mL	53–68 mL	64–77 mL	Maintained	Not available
SVR	Not reported	1100–1200 dynes·sec/cm^5^	741–897 dynes·sec/cm^5^	Improved	Not available
RA pressure	15 mmHg	Not recorded	Decreased trend (CVP 1–5 mmHg)	Stable	Not available
RV pressure	62/36 mmHg	Not available	Not available	Not available	Mild–moderate RV dysfunction
Pulmonary artery pressure	67/39 mmHg (mean 49)	58–66/28–37 (mean 41–50)	47–53/20–22 (mean 33–38)	45/26 (mean 33)	Not available
Pulmonary capillary wedge pressure	40 mmHg	Not available	Not available	Not available	Not available
PAPI	1.86	Not available	Improved qualitatively	Not available	Not available
Mixed venous oxygen saturation	Not available	Not available	Improved to >90%	94%	Not available
Lactate	2.2 mmol/L	Peak 3.2 mmol/L then 3.0 → 2.9 mmol/L	Improved to 2.0–1.5 mmol/L	1.6 mmol/L	Not available
Urine output (24 h)	400–1250 mL/day before support	1550 mL	3450 mL	2745–1705 mL	Not available
Creatinine	Increased to 2.22 mg/dL (baseline 1.32)	1.40 mg/dL	1.30 mg/dL	1.11–1.28 mg/dL	Subsequent postcardiotomy shock
BUN	Peak 68 mg/dL	42–48 mg/dL	35 mg/dL	26–28 mg/dL	Not available
AST/ALT	Severe ischaemic hepatitis (AST peak 2459 U/L; ALT peak 1121 U/L)	Downtrending	Continued improvement	AST 149–365; ALT 62–200 U/L	Not available
Total bilirubin	Peak 2.0 mg/dL	2.3 mg/dL	2.8 mg/dL	3.9–4.9 mg/dL	Not available
Complications	Cardiogenic shock with respiratory failure	None during support	None during support	None during support	Severe postcardiotomy LV dysfunction requiring VA-ECMO and Impella CP

On hospital day 9, after achieving relative clinical stabilization, he underwent a redo sternotomy. The decision to undergo surgical redo valve replacement was mainly considered owing to other concomitant valvular disease and ascending aortic aneurysm.

The patient underwent complex surgical procedures, including redo AVR with a 27 mm Inspiris bioprosthetic valve, mitral valve repair involving triangular resection of segment P2 and posterior band annuloplasty with a 34 mm Cosgrove ring, and wrapping of the ascending aortic aneurysm, reducing its size from 4.5 to 3 cm. The patient’s recovery was long and complicated and required escalation to VA-ECMO and Impella CP, and he went to advanced heart failure therapies at a different institution.

## Discussion

We present two complex cases of severe bioprosthetic AR complicated by cardiogenic shock, successfully managed through the use of MCS with Impella 5.5 as a bridge to definitive interventions: ViV TAVR in one case, and redo SAVR with additional mitral valve and aortic procedures in the other (*Table 3*). These cases highlight the novel utility of Impella 5.5 in the context of acute bioprosthetic valve failure.

**Table 3:** Comparison of baseline characteristics and outcomes between the two cases

Demographic characteristics, case presentation, and outcomes		
	Case 1	Case 2
Age	74	63
Sex	Female	Male
History of bicuspid aortic valve	No	Yes
Valvular pathology	Bioprosthetic severe AR	Bioprosthetic severe AR and severe mitral regurgitation
Urgency of procedure	Urgent	Urgent
Method of intervention	ViV transcatheter aortic valve replacement	Redo sternotomy with aortic valve replacement and mitral valve repair with ascending aorta wrap
Procedural complication	Complete heart block requiring pacemaker.	Cardiogenic shock and left ventricular dysfunction
Mechanical circulatory support	Impella 5.5	Impella 5.5, escalated to VA-ECMO and impella CP.
Outcome	Discharge to cardiac rehabilitation, complete recovery in NYHA Class I	Discharge to advanced heart failure facility

Management of severe AR complicated by cardiogenic shock remains particularly challenging because available temporary MCS options may worsen underlying valve physiology. IABP is generally avoided because augmentation during diastole may increase regurgitant volume, while VA-ECMO can increase afterload and left ventricular distension if unloading is inadequate.^1,2^ Impella support in severe AR has also traditionally been considered contraindicated or relatively contraindicated because forward flow generated into the ascending aorta may partially recirculate across the incompetent aortic valve back into the left ventricle, potentially worsening LV volume overload and limiting effective systemic output.^3^

Despite this concern, Impella 5.5 was selected in both cases after multidisciplinary heart team discussion because alternative MCS strategies were felt to carry comparable or greater physiological limitations and definitive valve intervention could not be performed immediately. Importantly, the intention was not durable support but short-term stabilization and bridge-to-definitive therapy.

Several factors supported continued use after implantation. First, serial invasive haemodynamic measurements demonstrated objective evidence of effective unloading rather than worsening regurgitant physiology. In Case 1, Impella support was associated with preservation of cardiac output and cardiac index, reduction in filling pressures, improvement in PAPs, increase in urine output, lactate normalization, and recovery of renal function before definitive intervention. In Case 2, invasive monitoring demonstrated improvement in cardiac output (3.1 to >6 L/min), cardiac index (1.7 to ∼2.9 L/min/m^2^), reduction in pulmonary pressures, improved end-organ perfusion, and renal recovery while supported.

Second, the risk of clinically significant recirculation was assessed continuously using a combination of invasive haemodynamics, laboratory markers of perfusion, and echocardiographic assessment rather than valve severity alone. Device position was confirmed at implantation and monitored serially with echocardiography to maintain appropriate inflow position within the left ventricle and avoid interaction with prosthetic valve structures. Effective unloading was inferred by improvement in cardiac performance and end-organ markers as well as reduction in left-sided filling pressures and downward trends in LV filling indices (including left ventricular end-diastolic pressure when available), rather than by complete elimination of regurgitant flow.

These findings align with emerging reports suggesting that severe AR may represent a relative rather than absolute contraindication to Impella 5.5 in carefully selected patients when support duration is short, definitive intervention is planned, and close haemodynamic and imaging surveillance is maintained.^3–10^ However, our cases should not be interpreted as evidence that Impella reliably overcomes regurgitant physiology. Case 2 in particular illustrates that despite successful preoperative stabilization, overall outcomes remained dependent on the underlying myocardial reserve and complexity of surgical intervention, ultimately requiring escalation to VA-ECMO and advanced heart failure support.

Accordingly, we view Impella 5.5 not as definitive therapy for severe AR, but as a potential bridge strategy in selected patients where objective evidence of unloading can be demonstrated and definitive valve intervention is achievable within a short timeframe.

Overall, our experience reinforces the emerging clinical consensus advocating for early use of Impella 5.5 as a strategic bridge to valve replacement therapies in patients with acute bioprosthetic AR and cardiogenic shock. Future multicentre studies are warranted to validate these findings, optimize patient selection, and determine the most effective bridging protocols.

## Conclusion

Impella 5.5 may represent a feasible bridge-to-definitive therapy strategy in selected patients with severe bioprosthetic AR and cardiogenic shock. In this small case series, support was associated with temporary haemodynamic stabilization and improved end-organ perfusion, enabling definitive intervention; however, outcomes remained heterogeneous, including the need for escalation to advanced mechanical support. Larger studies are required before conclusions regarding safety, effectiveness, or generalizability can be made.

## Supplementary Material

ytag573_Supplementary_Data

## Data Availability

The authors have no additional data to provide.

## Lead author biography

Hussam Al Hennawi is a cardiology fellow at Geisinger Wyoming Valley Hospital. His clinical and academic interests include interventional cardiology and structural heart disease.

## Notes

**Consent:** The authors confirm that written consent for submission and publication of this case report, including images and associated text, has been obtained from the patient, in line with COPE guidance.
