# Supplementary material for: Clinical applications of Impella 5.5 for acute severe bioprosthetic aortic regurgitation: a bridge to destination therapy report—case series and review
Source: Eur Heart J Case Rep. 2026 Aug 3;10(8):ytag573. doi: 10.1093/ehjcr/ytag573 (PMC13463636; doi:10.1093/ehjcr/ytag573)
Supplement: Supplementary file 1 [file ytag573_supplementary_data.zip › Supplemental video legends.docx]

**Videos**

Video 1. A transesophageal echocardiogram showing severe bioprosthetic aortic regurgitation.

Video 2. A transesophageal echocardiogram showing severe bioprosthetic aortic regurgitation.

Video 3: Fluoroscopy displays the transcatheter valve-in-valve procedure under angiographic guidance after retracting the Impella device into the ascending aorta.

Video 4. Post-procedural transesophageal echocardiogram showing successful ViV deployment without valvular or paravalvular leaks.

Video 5. A transesophageal echocardiogram showed a flail posterior leaflet with a ruptured chord.

Video 6. Transesophageal echocardiogram showing severe bioprosthetic aortic regurgitation.
